# Supplementary figures and images for: Tadr is an axonal histidine transporter required for visual neurotransmission in Drosophila
Source: eLife. 2022 Mar 1;11:e75821. doi: 10.7554/eLife.75821 (PMC8916773; doi:10.7554/eLife.75821)

Figure 2-figure supplement 2 source data 1

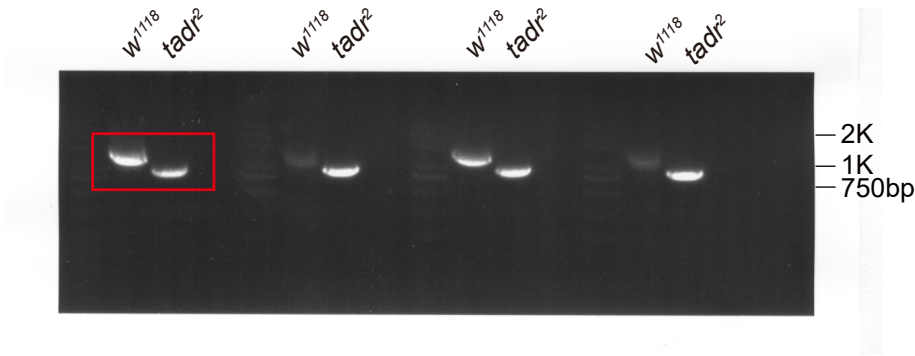

Supplement: Figure 2—figure supplement 2—source data 1. [file elife-75821-fig2-figsupp2-data1.pdf]

Figure 4-figure supplement 1 source data 1

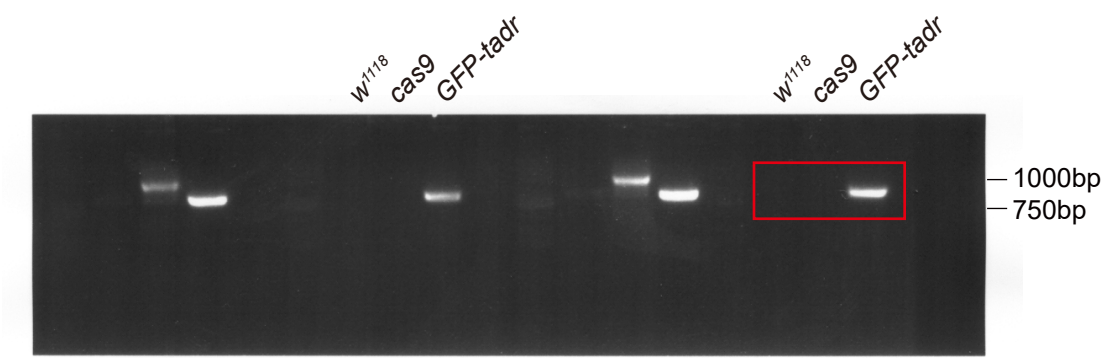

Supplement: Figure 4—figure supplement 1—source data 1. [file elife-75821-fig4-figsupp1-data1.pdf]
